# Supplementary material for: Tree crickets optimize the acoustics of baffles to exaggerate their mate-attraction signal
Source: eLife. 2017 Dec 11;6:e32763. doi: 10.7554/eLife.32763 (PMC5764570; doi:10.7554/eLife.32763)
Supplement: Transparent reporting form [file elife-32763-transrepform.pdf]

1st Floor
24 Hills Road
Cambridge CB2 1JP, UK

P 01223 855340
W elifesciences.org
T @elife

eLife's transparent reporting form

We encourage authors to provide detailed information *within their submission* to facilitate the interpretation and replication of experiments. Authors can upload supporting documentation to indicate the use of appropriate reporting guidelines for health-related research (see EQUATOR Network), life science research (see the BioSharing Information Resource), or the ARRIVE guidelines for reporting work involving animal research. Where applicable, authors should refer to any relevant reporting standards documents in this form.

If you have any questions, please consult our Journal Policies and/or contact us: editorial@elifesciences.org.

Sample-size estimation

- You should state whether an appropriate sample size was computed when the study was being designed
- You should state the statistical method of sample size computation and any required assumptions
- If no explicit power analysis was used, you should describe how you decided what sample (replicate) size (number) to use

Please outline where this information can be found within the submission (e.g., sections or figure legends), or explain why this information doesn't apply to your submission:

eLife Sciences Publications, Ltd is a limited liability non-profit non-stock corporation incorporated in the State of Delaware, USA, with company number 5030732, and is registered in the UK with company number FC030576 and branch number BR015634 at the address 1st Floor, 24 Hills Road, Cambridge CB2 1JP | August 2014

We do not use explicit power analyses in our manuscript since they do not apply to the information we wish to collect. There are three types of data in our manuscript, simultaneous vibrometry & sound recording, finite element (FE) models and behavioural data.

The data from vibrometry and sound recording have high accuracy and are very repeatable within each measurement (per animal). We are interested in the constant that describes the relationship between vibration levels and sound levels, a well understood physical phenomenon. Given the high accuracy of measurement and a deterministic mechanical relationship between the measured quantities, a small sample size is reasonable and a power analysis is not required.

Similarly, the FE models are based on deterministic not stochastic relationships between variables and replicating models is not relevant. A sensitivity analysis is relevant, was done and is mentioned in the supplementary methods in the supplementary section (Element details).

There are data from three behavioural experiments and in each of these experiments, we use very large replicates by the standards of the field. In the field experiment, we determined the period over which the measurement was made (3 field seasons) and the replicate size, animal number, was determined by the number of animals calling in the field site.

In the single leaf experiment, we used 51 animals which is a very large sample and is adequate for judging the probability of baffling.

In the choice experiment, we needed to judge whether the animals selected the larger leaf above chance (i.e. 50%) and an N of 15 was determined to be sufficient for this. The experiment was performed on 19 males, of which 15 sang and data were collected only from these males.

To estimate the natural distribution of leaf sizes, we chose to randomly sample 5 leaves from a very large number of plants which males were calling from (N=114). The total sample size of leaves achieved (N=570) is sufficient to generate a leaf size distribution that is representative of the plants used by calling tree crickets.

Replicates

- You should report how often each experiment was performed
- You should include a definition of biological versus technical replication
- The data obtained should be provided and sufficient information should be provided to indicate the number of independent biological and/or technical replicates
- If you encountered any outliers, you should describe how these were handled
- Criteria for exclusion/inclusion of data should be clearly stated

eLife Sciences Publications, Ltd is a limited liability non-profit non-stock corporation incorporated in the State of Delaware, USA, with company number 5030732, and is registered in the UK with company number FC030576 and branch number BR015634 at the address 1st Floor, 24 Hills Road, Cambridge CB2 1JP | August 2014

- High-throughput sequence data should be uploaded before submission, with a private link for reviewers provided (these are available from both GEO and ArrayExpress)

Please outline where this information can be found within the submission (e.g., sections or figure legends), or explain why this information doesn't apply to your submission:

The vibrometry & sound recording experiment was biologically replicated 5 times, i.e. performed on 5 animals. This sample size is mentioned in the results section (Calling effort and wing speed) and in the supplementary methods (Vibration measurement of singing crickets in the laboratory). Technical replication involved taking a sample that was 1.024 seconds in length, corresponding to ~3100 cycles of the call frequency, which allowed us to collect data from several syllables of call and this is mentioned in the supplementary methods (Vibration measurement of calling crickets in the laboratory.)

The behavioural experiments all have only biological replications.

The field behavioural experiment was performed over three field seasons and the data on leaf length was pooled across the seasons, thus giving us a replicate size of 27 baffling leaves and 78 non-baffling leaves. The replicate size is mentioned in the supplementary methods (Baffling and non-baffling leaf size measurements in the wild) and in the figure S3 itself. Outliers are excluded only for plotting using Tukey's method (above or below 1.5 x interquartile range). For the Mann-Whitney U test all data included.

The single leaf experiment was performed in a repeated presentation paradigm on 51 individuals and this replicate size is mentioned in the supplementary section (Effect of leaf size on likelihood of baffling).

The two-leaf choice experiment was performed on 19 males, of which 15 males called. No data were collected from 4 males that did not call. This replicate size of 15 is mentioned in the results section (Optimization of baffle acoustics) and in the supplementary section (Leaf size preference and creation of a baffle hole).

Statistical reporting

- Statistical analysis methods should be described and justified
- Raw data should be presented in figures whenever informative to do so (typically when N per group is less than 10)
- For each experiment, you should identify the statistical tests used, exact values of N, definitions of center, methods of multiple test correction, and dispersion and precision measures (e.g., mean, median, SD, SEM, confidence intervals; and, for the major substantive results, a measure of effect size (e.g., Pearson's r, Cohen's d)
- Report exact p-values wherever possible alongside the summary statistics and 95% confidence intervals. These should be reported for all key questions and not only when the p-value is less than 0.05.

Please outline where this information can be found within the submission (e.g., sections or figure legends), or explain why this information doesn't apply to your submission:

The first statistical test in the manuscript is the Mann-Whitney U test used to test whether the lengths of leaves used by males for baffled versus non-baffled calling were different. All details are given in the supplementary section (Baffling and non-baffling leaf size measurements in the wild) and the legend of figure S3.

The other set of statistical tests are the Cochran's Q test used to test whether baffling probability changed with leaf size and McNemar's tests used for post-hoc comparison. All details are available in the main MS itself and in the supplementary materials.

All other statistical representations are either proportions or representations of central tendencies, in which we always indicate that the measure is mean ± SE and N as appropriate.

(For large datasets, or papers with a very large number of statistical tests, you may upload a single table file with tests, Ns, etc., with reference to sections in the manuscript.)

Group allocation

- Indicate how samples were allocated into experimental groups (in the case of clinical studies, please specify allocation to treatment method); if randomization was used, please also state if restricted randomization was applied
- Indicate if masking was used during group allocation, data collection and/or data analysis

Please outline where this information can be found within the submission (e.g., sections or figure legends), or explain why this information doesn't apply to your submission:

This information does not apply to our manuscript since we did not allocate samples to experimental groups.

In the single leaf behavioural experiment, a repeated exposure design was used and all animals were in all groups.

In the field behavioural experiment, the animals chose the baffled or non-baffled condition themselves.

Additional data files ("source data")

- We encourage you to upload relevant additional data files, such as numerical data that are represented as a graph in a figure, or as a summary table

- Where provided, these should be in the most useful format, and they can be uploaded as “Source data” files linked to a main figure or table
- Include model definition files including the full list of parameters used
- Include code used for data analysis (e.g., R, MatLab)
- Avoid stating that data files are “available upon request”

Please indicate the figures or tables for which source data files have been provided:

We have submitted our data to Dryad and the temporary link (not to be used in the final MS as per Dryad instructions) is as follows: http://datadryad.org/review?doi=doi:10.5061/dryad.f9011

eLife Sciences Publications, Ltd is a limited liability non-profit non-stock corporation incorporated in the State of Delaware, USA, with company number 5030732, and is registered in the UK with company number FC030576 and branch number BR015634 at the address 1st Floor, 24 Hills Road, Cambridge CB2 1JP | August 2014
